# Economic Model for Cost Containment: Evaluating the Utilization of a Touch-free Zinc Oxide in an Acute Care Hospital

**DOI:** 10.7759/cureus.4957

**Published:** 2019-06-20

**Authors:** Ana Clara Tolentino, Sonya Dick

**Affiliations:** 1 Gerontology/wound and Ostomy, Universidade do Estado do Rio de Janeiro, Rio de Janeiro, BRA; 2 Neuroscience, University of Kentucky College of Medicine, Bowling Green, USA

**Keywords:** dermatitis, zinc oxide, treatment outcome, hospital costs

## Abstract

Introduction

Incontinence-associated dermatitis (IAD), the most common form of moisture-associated skin damage (MASD), puts patients at higher risk for ulceration. Treatment of MASD/IAD includes application of zinc oxide, typically applied using gloved hands directly to the area of concern. A computational model was utilized to examine a cost comparison of a touch-free zinc oxide treatment versus traditional zinc oxide for MASD/IAD.

Methods

A literature search was performed using PubMed, a nursing journal database (CINAHL), and MEDLINE for publications from January 1, 2010 through November 30, 2017. Data on prevalence of MASD/IAD, average length of stay, and time to heal were extracted and utilized in the computational model. Cost per patient stay and annual total hospital costs were calculated for three and four applications of zinc oxide per patient with an averaged prevalence rate of 25% for a hypothetical hospital.

Results

The computational model estimated a range of cost savings between $181.88 to $2,000.63 per patient stay, and $4,728.88 to $52,016.25 over a 12-month period compared to traditional zinc oxide application.

Conclusions

The computational model estimated a cost savings of up to $52,016.25 per year in a hypothetical 250-bed acute care hospital compared to traditional zinc oxide application. Future prospective studies examining clinical effectiveness and health economics of touch-free zinc oxide are necessary.

## Introduction

Incontinence-associated dermatitis (IAD), the most common form of moisture-associated skin damage (MASD), is a major concern with hospitalized patients and puts them at higher risk for skin breakdown and ulceration [1-3]. Zinc oxide has traditionally been utilized to prevent and treat MASD/IAD [4,5]. Zinc oxide ointments have been applied with gloved hands directly to the area of concern, often the perianal and perineal area of patients.

The rising costs of healthcare, especially those related to the length of stay and chronic conditions, have led to the development of products focused on maximizing patient care while minimizing costs. Recently, a touch-free zinc oxide product has become commercially available that allows for improved application through a touch-free spray requiring a reduced amount of zinc oxide needed for complete coverage. In order to examine if the touch-free zinc oxide provides cost savings, a hypothetical computer model was used to compare potential costs resulting from the utilization of touch-free zinc oxide to traditional zinc oxide ointment to treat MASD/IAD in an acute care patient population.

## Materials and methods

This work was previously presented at Symposium on Advanced Wound Care Fall (Poster: Dick S, Tolentino AC. Economic Model for Cost Containment: Evaluating the Utilization of a Touch-Free Zinc Oxide in an Acute Care Hospital. Symposium on Advanced Wound Care Fall; November 2-4, 2018).

Literature search

A literature search was performed using PubMed, a nursing journal database (CINAHL), and MEDLINE for publication dates of January 1, 2010 through November 30, 2017. The following search terms were used: “incontinence associated dermatitis” or “moisture associated skin damage” or “IAD” or “MASD” and “Prevalence Rate” or “Incidence Rate” and “Length of Stay” or “Time to Heal” or “LOS” or “TTH”. Studies with the following criteria were utilized for data collection: abstract or manuscript written in English, published study or peer-reviewed conference proceeding, clinical sites including acute care hospital and long-term acute care facilities. Studies with the following criteria were excluded: preclinical studies (animal or bench studies), pediatric patient population, studies without methodological data collection standards. Four studies were found to meet the criteria. Data on prevalence of MASD/IAD, average length of stay (LOS) and time to heal (TTH) were extracted from the published articles and utilized in the computational model.

Computational model

A non-validated computational model based on data obtained for prevalence of MASD/IAD, LOS and TTH was created to estimate the potential cost of care (Poster: Marxen A, Jackson S, Stephenson C. An Assessment of the Cost Benefits of Zinc Oxide Spray vs. Tube Application. Wound Ostomy and Continence Nurses Society; May 19-23, 2017; Poster: Milne CT. Hands-Off! Using a Spray Application Delivery System to Impact Bacterial Contamination of Moisture Barriers. Wound Ostomy and Continence Nurses Society; June 4-8, 2016). Application of a touch-free zinc oxide treatment (Touchless Care® Zinc Oxide, Crawford Healthcare Limited, an ACELITY Company, Doylestown, PA) versus direct gloved application of zinc oxide ointment was compared per group of patients (n = 62.5) at an averaged prevalence rate of 25% (Poster: Junkin J, Moore-Lisi G, Selekof JL. What We Don’t Know Can Hurt Us: Pilot Prevalence Survey of Incontinence and Related Perineal Skin Injury in Acute Care. Clinical Symposium on Advances in Skin and Wound Care; October 23-26, 2005) [6]. Average unit costs per product were included in the analysis and calculated for a 250-bed-acute care hypothetical hospital. It was estimated that the touch-free zinc oxide spray could have a maximum of 51 applications per 2 oz bottle (estimated from four sprays per application and ~205 sprays per bottle, per product insert); while one tube of zinc oxide ointment was estimated to have between four and 12 applications per 4 oz tube (estimate obtained from 12 polled health care providers [HCPs]).

The range of zinc oxide applications within a 24-hour period of patient care used in the computational model was obtained from 12 polled HCPs. The HCPs were from an acute care setting, including hospitals, and long-term acute care settings. They consisted of five wound ostomy continence nurses, four nurse practitioners, and three physical therapists with Clinical Wound Specialist certification. The HCPs were polled at random during the Symposium on Advanced Wound Care conference (October 20-22, 2017) with a simple question of “On average, how often do you apply or reapply zinc oxide for MASD/IAD during a 24-hour period per patient at your facility?” The options given were one to two times, three to four times or five to six times. Results for polled responses included one to two applications (n = 2), three to four applications (n = 8) and five to six applications (n = 2). As most polled HCPs estimated three to four applications per patient per day of zinc oxide at their facilities, three to four applications were incorporated into the computational model. Unit pricing for zinc oxide ointment and touch-free zinc oxide spray was obtained from an average tier pricing program of a national group purchasing organization in 2018.

## Results

Data from four studies obtained from the literature search were utilized for the computational model (Poster: Junkin J, 2005) [6-8]. Prevalence of MASD/IAD was reported as 20% and 27%, respectively, in two acute care studies (Poster: Junkin J, 2005) [6]. The average LOS for MASD/IAD patients was reported to be 15 days [7], while the reported TTH was 11 days [8]. A prevalence of 25% of MASD/IAD, an LOS of 15 days, and a TTH of 11 days were estimated for the computational model. The cost per patient stay and annual total costs were calculated for three and four applications per day of zinc oxide based on the polled HCP responses and four application per tube of zinc oxide ointment (Tables 1-4). Given a prevalence of 25%, approximately 62.5 patients in a 250-bed hospital wound develop MASD/IAD. Additionally, given an average LOS of 15 days, the patient stay costs were calculated per a two-week period to get final annualized incremental costs. There was a range of cost reduction with touch-free zinc oxide use from $26.92 to $32.01 per individual patient stay (Tables 1, 3) and from $1,682.34 to $2,000.63 per all patient stays (Tables 2, 4). An annual cost reduction range of $43,745.00 to $52,016.25 over a 12-month or 1-year period for a hypothetical 250-bed Hospital XYZ was calculated for the touch-free zinc oxide use compared to traditional zinc oxide use (Tables 2, 4). When an estimate of 12 zinc oxide ointment applications per tube is used, a range of cost reductions from $2.91 to $5.82 per individual patient stay and $181.88 to $363.75 per all patients stays is calculated. An annual cost reduction range $4,728.88 to $9,457.50 over a 12-month or one-year period for a hypothetical 250-bed Hospital XYZ was calculated for the touch-free zinc oxide.

**Table 1:** Per patient costs of zinc oxide usage with three applications per day with four applications per tube of ointment LOS: Length of stay; MASD: Moisture-associated skin damage; IAD: Incontinence-associated dermatitis; US: United States. ( ) denotes savings with touch-free zinc oxide. *Cost calculated by subtracting the cost of zinc oxide ointment from the cost of touch-free zinc oxide.

	Average LOS MASD/IAD	Zinc Oxide Applications Per Day	Total Zinc Oxide Applications Per LOS	Applications Per Full Bottle/Tube	Number of Bottles/Tubes Per Patient	Cost Per Unit (US $)	Cost Per Patient Stay (US $)
Zinc Oxide Ointment	15	3	45	4	11.25	$2.91	$32.74
Touch-free Zinc Oxide	15	3	45	51	1	$5.82	$5.82
				Cost*			($26.92)

**Table 2:** Annual total hospital costs for three applications per day of zinc oxide usage MASD: Moisture-associated skin damage; IAD: Incontinence-associated dermatitis; TTH: Time to heal; US: United States. *ONLY direct costs related to the product itself have been considered, according to the prevalence rate. ( ) denotes savings with touch-free zinc oxide.

	Number of Acute Care Beds	MASD/IAD Prevalence (n = 62.5)	MASD/IAD TTH (Days)	Cost Per Patient Stay* (US $)	Total Patient Costs Per Stay* (US $)	Annualized Treatment Costs* (US $)
Zinc Oxide Ointment	250	25%	11	$32.74	$2,046.25	$53,202.50
Touch-free Zinc Oxide	250	25%	11	$5.82	$363.75	$9,457.50
	Incremental costs				($1,682.50)	($43,745.00)

**Table 3:** Calculated cost of zinc oxide per patient stay with four applications per day with four applications per tube of ointment LOS: Length of stay; MASD: Moisture-associated skin damage; IAD: Incontinence-associated dermatitis; US: United States. ( ) denotes savings with touch-free zinc oxide. *Cost calculated by subtracting the cost of zinc oxide ointment from the cost of touch-free zinc oxide.

	Average LOS for MASD/IAD	Zinc Oxide Applications Per Day	Total Zinc Oxide Applications Per LOS	Applications Per Full Bottle/Tube	Number of Bottles/Tubes Per patient	Cost Per Unit (US $)	Cost Per Patient Stay (US $)
Zinc Oxide Ointment	15	4	60	4	15	$2.91	$43.65
Touch-free Zinc Oxide	15	4	60	51	2	$5.82	$11.64
				Cost*			($32.01)

**Table 4:** Annual total hospital costs of zinc oxide usage with four applications per day MASD: Moisture-associated skin damage; IAD: Incontinence-associated dermatitis; TTH: Time to heal; US: United States. *ONLY direct costs related to the product itself have been considered, according to the prevalence rate. ( ) denotes savings with touch-free zinc oxide.

	Number of Acute Care Beds	MASD/IAD Prevalence (n = 62.5)	MASD/IAD TTH (Days)	Cost Per Patient Stay* (US $)	Total Patient Costs Per Stay* (US $)	Annualized Treatment Costs* (US $)
Zinc Oxide Ointment	250	25%	11	$43.65	$2,728.13	$70,931.38
Touch-free Zinc Oxide	250	25%	11	$11.64	$727.50	$18,915.00
	Incremental Costs				($2,000.63)	($52,016.38)

## Discussion

As healthcare costs are increasing, healthcare centers are prioritizing treatments that reduce costs and hospital LOS. Due to the prevalence, cost, and time of care associated with MASD/IAD, treatment for this condition is one area for potential cost savings [9]. A touch-free zinc oxide application system has been developed that may help reduce overall healthcare costs associated with prevention and treatment of MASD/IAD. A computational model was developed to estimate the cost of using touch-free zinc oxide compared to traditional zinc oxide and indicated a potential annual product cost savings of up to $52,016.25 in a hypothetical 250-bed acute care hospital. These calculations were purely based on product costs. Further savings may be realized with factoring in nursing time, glove or unused product, and the potential reduction of contamination of product containers.

Traditionally, MASD/IAD is treated with the application of zinc oxide ointment or cream [4,5]. However, this requires the use of gloves and multiple zinc oxide applications to ensure adequate coverage, increasing nursing time per patient and the use of consumables. Additionally, the traditional zinc oxide ointments are packaged in tubes, making it difficult to fully expel all the product out of the tube, increasing product waste. The touch-free zinc oxide application is performed using a spray technique, reducing the need for multiple glove changes per application and leaving less product waste. Marxen et al. compared glove and product waste following three zinc oxide application methods (spray and two ointments) (Poster: Marxen A, Jackson S, Stephenson C. An Assessment of the Cost Benefits of Zinc Oxide Spray vs. Tube Application. Wound Ostomy and Continence Nurses Society; May 19-23, 2017). Marxen et al. noted 0.31 oz to 0.66 oz of glove waste and 0.44 oz to 0.90 oz of product waste following traditional zinc oxide application compared to 0.2 oz of bottle waste associated with the touch-free zinc oxide application (Poster: Marxen A, May 19-23, 2017). These results indicate that in terms of consumables and product waste, the use of touch-free zinc oxide has the potential to reduce costs further when consumables are taken into consideration.

Product ease of use and removal can also affect healthcare costs by altering the amount of time needed for patient care. A product that is easy to apply and remove will take less nursing time than a more complex application and removal. A study examining ease of application and removal indicated that compared to traditional zinc oxide ointments, touch-free zinc oxide was rated as easier to apply and remove by HCPs (Poster: Roberts S, Warde D, Marxen A, Thompson J, Stephenson C. A Comparison of the Application, Removal, and Cost Effectiveness of Zinc Oxide Barrier Products. Wild on Wounds; September 2-5, 2015). While ease of use was examined, the time needed for application and removal was not measured. However, these results indicate that the touch-free zinc oxide application and removal have the potential to reduce nursing time needed for treatment application and removal for MASD/IAD patients.

In patients with IAD, there exists an increased potential for bacterial contamination of the product packaging and the development of infection among patients via direct contact of the treatment location. The costs of treating infections can be expensive and drive up healthcare costs. In comparing traditional zinc oxide ointment with the touch-free zinc oxide spray application after seven days of clinical use, the spray application showed reduced bacterial growth in cultures from the product opening after seven days of clinical use (Poster: Milne CT. Hands-Off! Using a Spray Application Delivery System to Impact Bacterial Contamination of Moisture Barriers. Wound Ostomy and Continence Nurses Society; June 4-8, 2016). This indicates that the touch-free zinc oxide may help mitigate the reintroduction of bacteria to patients with MASD/IAD.

Limitations exist for this study. A literature search was employed to identify the prevalence of MASD/IAD in acute care settings as well as average TTH and LOS for this hypothetical patient population. However, a limited amount of literature-based evidence exists. This is particularly due to the inappropriate use and interchanged use of the terms IAD and MASD in the published literature. To our knowledge, this is the first publication focusing on touch-free zinc oxide, limiting the ability to directly compare and contrast our health economic data with the currently published literature. In order to compare costs, we employed a hypothetical model of touch-free zinc oxide use or traditional zinc oxide use in MASD/IAD. This model while informative on the potential cost benefits of touch-free zinc oxide, might differ from real-world cost-effectiveness studies due to variables such as health insurance reimbursement rates, variations in MASD/IAD rates, and population differences. However, while the model is hypothetical, it can be adopted for real-world use and tailored to the needs of the healthcare setting.

## Conclusions

The computational model estimated a potential range of yearly product cost savings between $4,728.88 and $52,016.25 with the use of touch-free zinc oxide compared to traditional zinc oxide in a hypothetical 250-bed acute care hospital. The computational model can be adjusted with pricing, TTH, average LOS, hospital beds, and other variables which allow the hospital system to customize their calculations. Future prospective studies examining potential clinical effectiveness and health economics between touch-free zinc oxide to traditional zinc oxide creams in a patient population are necessary.
